# Vulcano island: the new high resolution digital surface model post 2021-2022 volcanic unrest

**DOI:** 10.1038/s41597-026-06623-7

**Published:** 2026-01-22

**Authors:** Marina Bisson, Roberto Gianardi, Francesca Iacono, Paolo Madonia, Gianfilippo De Astis, Claudia Spinetti

**Affiliations:** 1 Istituto Nazionale di Geofisica e Vulcanologia (INGV), Sezione Pisa, Pisa, Italy; 2 Dipartimento di Scienze della Terra, Università di Pisa, Pisa, Italy; 3 Istituto Nazionale di Geofisica e Vulcanologia (INGV), Sezione Osservatorio Etneo, Catania, Italy; 4 Istituto Nazionale di Geofisica e Vulcanologia (INGV), Sezione Roma 1, Roma, Italy; 5 Istituto Nazionale di Geofisica e Vulcanologia (INGV), Sezione Osservatorio Nazionale Terremoti, Roma, Italy

**Keywords:** Volcanology, Geomorphology, Natural hazards

## Abstract

During the years 2021-2022 Vulcano island was affected by a volcanic crisis characterized by a remarkable increase in fumarolic activity, intensified seismicity and ground deformations. With the aim of reproducing with high detail the island surface after this crisis, an Airborne Lidar survey was carried out on 4^th^ August 2023. More than 200 × 10^6^ 3D Lidar points were processed to create a new Digital Surface Model at a very high spatial resolution (50 cm). This model reproduces the elevation surface of all natural and anthropic elements constituting the island. The model was validated through a set of Ground Control Points and a vertical accuracy of 8 cm was obtained. This level of accuracy, combined with the spatial resolution of 50 cm, makes the model particularly suitable for detailed geomorphological investigations. In addition, associated with the derived coastline, it provides the most up-to-date and accurate 3D topography for the assessment of natural hazards, such as changes in the volcanic activity state, earthquakes and mass movements triggered by extreme rainfalls.

## Background & Summary

Updates of Digital Surface Model (DSM) are particularly relevant in active volcanic areas where landscape modifications are frequent. The comparison between DSMs generated before and after either effusive and/or explosive eruptions allows to map and quantify, both in terms of area and volume, the emplaced volcanic products as well as secondary phenomena, such as lahars or landslides. For quiescent volcanic areas, especially if populated, an updated and high spatial resolution topography can play an important role when the area is affected by a possible volcanic unrest, as occurred at Vulcano island during 2021-2022. In fact, during volcanic unrests, several landscape modifications can occur^1,2^.

The surface reconstruction of volcanic areas is usually obtained by elaborating remote sensing data acquired with different techniques, according to the extent of the area itself. For wide areas (several km^2^), the topography is commonly reconstructed applying stereo photogrammetry technique to satellite data^3,4^, or interpolating Airborne Lidar (Light Detection and Ranging) elevation points with dedicated algorithms. For areas ≤1 km^2^, the topography is reconstructed applying the SfM (Structure from Motion) technique to images acquired from Unoccupied Aircraft Systems (UAS)^5,6^. These techniques and data allow to reproduce topographies covering a wide range of spatial extents (from tens of square meters to tens of square kilometres), with spatial and temporal resolution associated to accuracy at different scales.

**Fig. 1:**
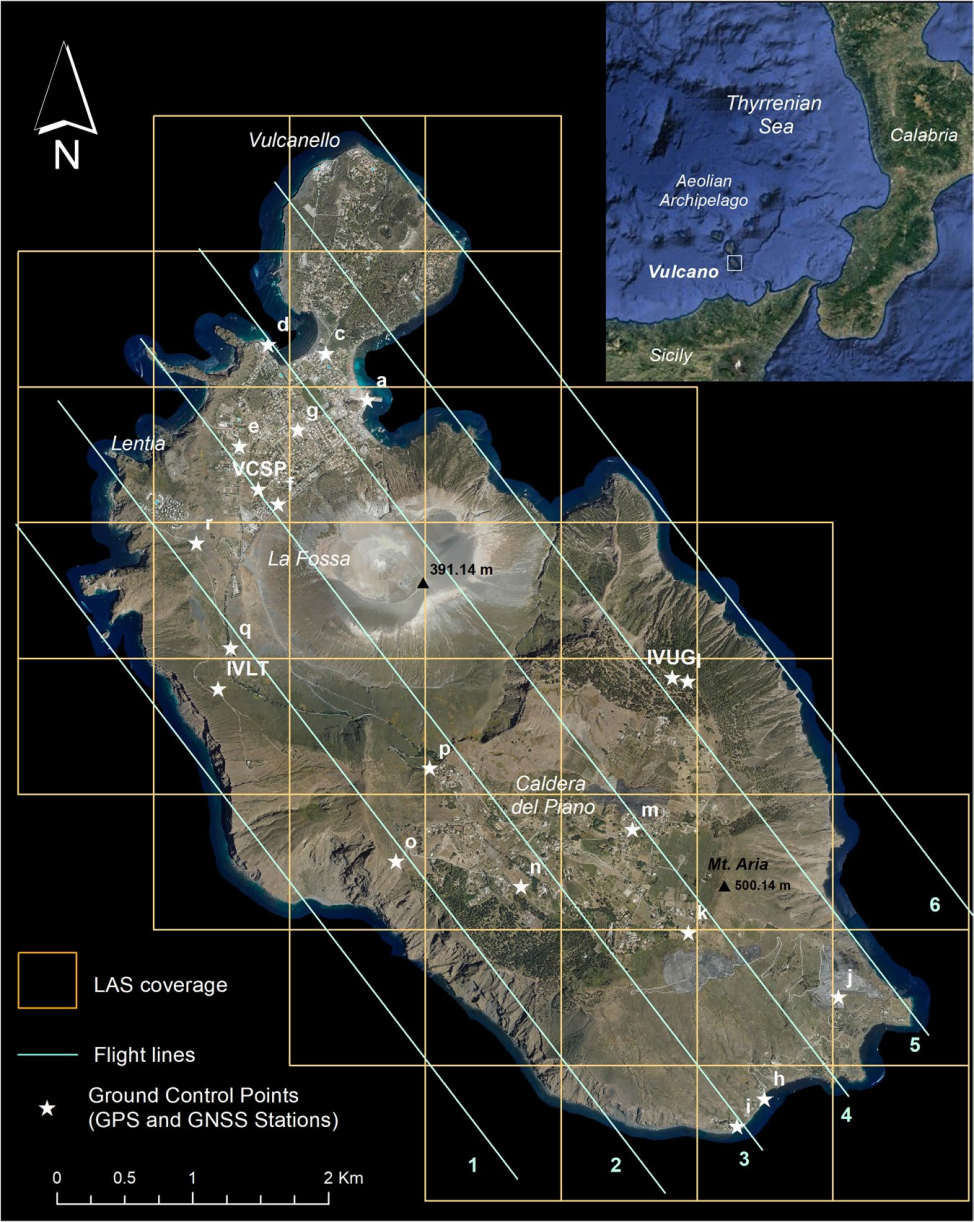
Map of Vulcano island. The Airborne Lidar flight lines, the LAS coverage and the locations of GCPs are visualized on the orthoimages at spatial resolution of 15 cm. The inset at the top right of the figure shows the geographical setting of the island.

Vulcano is the southernmost island of the Aeolian Archipelago, a volcanic arc located in the south-eastern sector of the Tyrrhenian Sea (Fig. 1) in correspondence of the subduction zone between the African and Eurasian plates.

The volcanic edifice consists of a subaerial portion that covers approximately 22 km², reaching the maximum elevation of 500 m a.s.l. at Mt. Aria^7^, and a submarine portion whose base lies at an average depth of 1 km b.s.l.^8^. The subaerial portion is the result of a volcanic activity started about 127 ka ago and described through eight main Eruptive Epochs stages^9,10^ that are represented through different volcanic centers: Paleo-Vulcano, Vulcano Primordiale and Piano Caldera (120-100 ka), Piano Caldera in-fill products and intra-caldera centers (100-20 ka), Lentia domes field (28-15 ka), La Fossa Caldera activity and volcanic units (15-8 ka), La Fossa Cone (from 5.5 ka to Present), and Vulcanello (183 B.C. to XVI sec.). Around 1550 AD, Vulcanello islet was connected to Vulcano by ash accumulation in the isthmus area. Among all these volcanoes, La Fossa Cone, located at the center of the La Fossa Caldera and reaching the maximum elevation of 391 m a.s.l., is currently considered an active composite edifice^11^. It has been characterized by an intense and continuous fumarolic activity since the last eruption dated 1888–1890^12^. This eruption, named “vulcanian” due to the peculiar characteristics if compared to all previously known eruptions^13^, was described in detail by Mercalli and Silvestri during their stay on the island on behalf of the Italian Government^12^. Different chrono-stratigraphic reconstructions have been provided for the La Fossa volcanic history^10,11,14,15^, with different implications for the assessment of the volcanic risk. However, they are beyond the scope of our work and therefore we will not address them. After the last eruption, Vulcano was affected by several unrests specifically during 1987–1993, 1996–1998 and 2004–2005^16^. All these episodes were characterized by significant changes in fumarole temperature and gas composition, generally accompanied by an increase in seismic-volcanic events and negligible ground deformations. Sixteen years after the previous unrest, a new one occurred in 2021. It started during the summer with remarkable changes in gas composition and temperature, increments of soil CO_2_ degassing and concentrations in fumaroles, accompanied by significant local seismicity and ground deformations^16–18^. These phenomena culminated in October 2021, creating very serious concerns to the population, with a “State of Emergency” declared by the Civil Protection Department^19^. The crisis lasted for more than 18 months, until summer 2023, when the Civil Protection Department officially declared the end of the ‘State of Emergency’, following a notable decrease of the unrest indicators.

With the aim to accurately reproduce the 3D topography of the island after the above described volcanic crisis, an Airborne Lidar survey was commissioned and carried out on 4^th^ August 2023. Millions of 3D points acquired during the flight were processed and elaborated to create a DSM at high spatial resolution, able to reproduce natural and anthropic features with elevated accuracy. This new and updated elevation model, compared with previous models available for the entire island^20–22^, or for some specific portions^23^, allows to identify and map natural and anthropic changes of the island during the last 30 years, providing a very useful tool for land management in case of natural calamities.

## Methods

### Data acquisition, processing and elevation surface generation

The Lidar photogrammetric survey was carried out on 4^th^ August 2023, using a Leica Geosystems Terrain Mapper System mounted on a Piper PA 31 aircraft, which flew over the entire island from 08:55 a.m. to 09:27 a.m. (local time) at an average elevation of 2,113 m a.g.l. (from 2,109 m to 2,117 m). The Leica Geosystems Terrain Mapper System used for this survey consists in the Hyperion 2 Lidar sensor and the Leica RCD30 photogrammetric camera (image sensor: 10,320 × 7,752 pixels), completed by an Inertial Measurement Unit (IMU) of latest generation, able of recording Global Navigation Satellite System (GNSS) Inertial measurements with high precision (https://leica-geosystems.com). In detail, during the 32 minutes of flight, Hyperion 2 sensor, operating in the Near Infrared Region (λ = 1.064 μm) with a scan angle of 36° and a pulse repetition frequency of 751 kHz, acquired a total of 220,886,253 elevation points. The planning assured an initial acquisition density of 10 points /m^2^, with an instrumental vertical error of ±5 cm (1 σ) and a planimetric error of ±13 cm (1 σ) for each acquired point. All points, acquired in ellipsoid heights and geo-localised in WGS 84 (ETRF2000) UTM 33 N thanks the IMU system, were stored and delivered in 39 LASer format (LAS) files (Fig. 1), each covering an area of 1 km × 1 km. Simultaneously with the acquisition of the Lidar points, a total of 174 photograms were captured along the six flight lines (Fig. 1), and processed obtaining 23 RGB orthoimages at a spatial resolution of 15 cm, stored in ECW format.

The processing of Lidar 3D points involved 3 main steps. The first step consisted in the conversion of the points elevations from ellipsoid to orthometric heights (ITALGEO 2005 geoid). This step was performed through the ConVERGO software that integrates the conversion grids (GK2) provided by the Istituto Geografico Militare Italiano (IGMI). The second step was aimed to identify outliers, spikes and double points (wrong points) through dedicated procedures performed on the TERRASOLID platform (www.terrasolid.com), and based on the analysis of echoes returns intensity and associated height values. In detail, Hyperion 2, the sensor used in this Airborne Lidar survey, is able to record up to 15 echoes (returns) per laser pulse. As the intensity of each echo is associated to the typology of the surface hit by the laser pulse, this sensor is able to classify a large variety of land surfaces. The third and final step of the processing involved the removal of the points identified as “wrong” by using specific filtering algorithms available on the TERRASOLID platform. The final points were 137,442,846, assuring an acquisition density of 6 points/m^2^. Such points were used for reconstructing the 3D surface of Vulcano island. This obtained surface is a DSM, as it includes the elevation of both natural (ground, vegetation) and anthropic features.

**Fig. 2:**
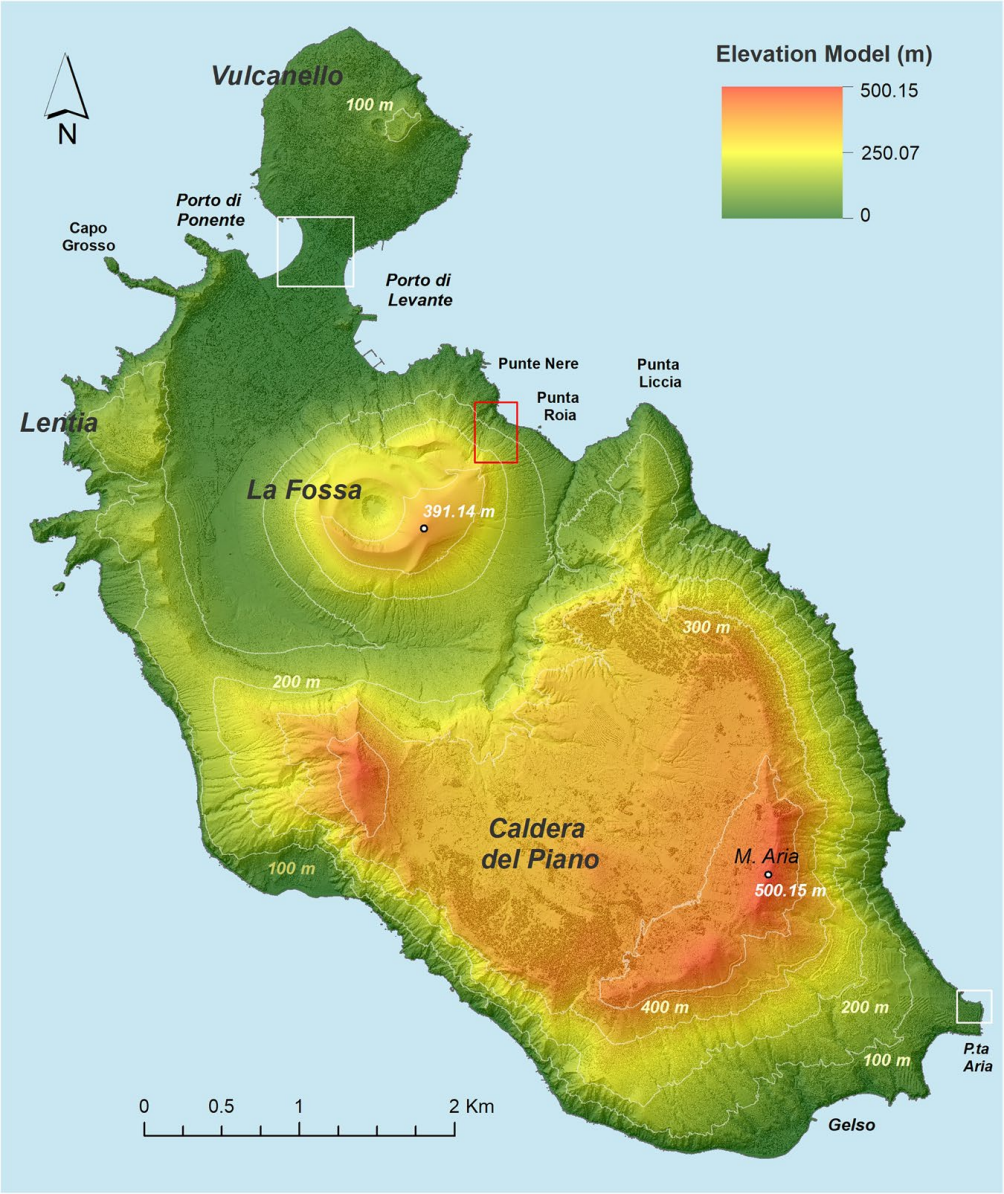
Digital Surface Model of Vulcano island. Elevation model is overlaid on the shaded relief map. The contour lines (in white) are reported every 100 meters.

The reconstruction of the DSM was firstly obtained by interpolating the final points by using the Delaunay algorithm^24^. Such interpolation produces a continuous vector 3D surface model named Triangular Irregular Network (TIN). Subsequently, the TIN model was transformed in a raster model with a 0.5 m × 0.5 m cell size. Such spatial resolution is consistent with the density of points available per square meter, and coherent with the use of Delaunay triangulation. These elaborations and the derived maps presented here (Elevation Model and Shaded Relief) were performed in the ESRI GIS environment. Figure 2 shows the resulting elevation model of Vulcano island overlapped to the shaded relief map derived from the DSM itself. The elevation of the model ranges from a sea level up to 500.15 m a.s.l. in correspondence with Mt. Aria, located in the southern part of the island. In the active area of La Fossa the maximum height (391.14 m a.s.l.) is reached on the southeaster edge of the Cone.

Thanks to further DSM elaborations and comparisons with the orthoimages datasets acquired during the same Airborne Lidar survey, it was possible to generate the vector data of the coastline. The high spatial resolution of the DSM (50 cm), associated with the higher spatial resolution of the orthoimages (15 cm), allowed the creation of a very detailed coastline of Vulcano island. This coastline is 43,999.97 m long, delimiting an emerging area of the island equal to 20,818,829.39 m^2^. In addition to the coastline, 465 emergent rocks with an area greater than 1 m² and 9 with an area smaller than 1 m² were individuated, providing a highly detailed shoreline dataset. This dataset can be useful for studies related to tsunami hazard scenarios, as those hypothesized during the recent crisis at Vulcano, and for multi-temporal analyses of coast evolution in relation to erosion and depositional dynamics and sea-level variations.

**Fig. 3:**
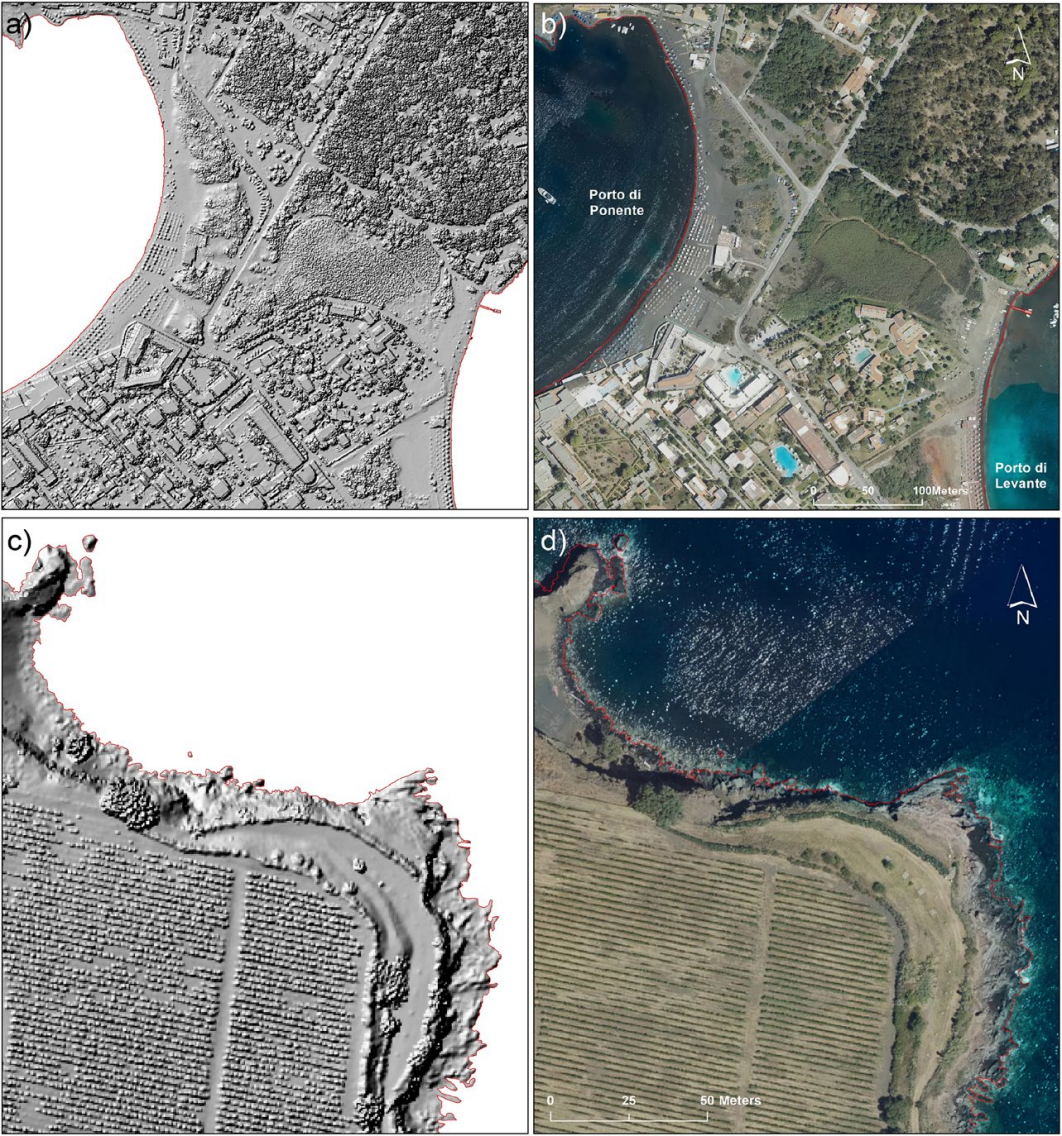
Shaded Relief derived from DSM and the relative orthoimages are shown for two selected areas. The first area is centred on the isthmus including the Porto di Ponente and a part of Porto di Levante (white inset in Figs. 2, 3a,b), the second area focuses on P.ta Aria. This promontory is situated east of Gelso locality (white inset in Figs. 2, 3c,d). Both selected areas show an accurate coastline (red line) derived from DSM.

The high detail of the DSM is shown in Fig. 3, comparing the Shaded Relief map and the relative orthoimages in two zones of the island characterized by different landscapes. The first zone, centred on the isthmus connecting Vulcanello to the main island (northern white inset of Fig. 2), is representative of a flat terrain with a mix of vegetated and anthropic areas (Fig. 3a,b). The DSM accurately reproduces all natural and human-made features visible in the orthoimage, even including beach umbrellas at Porto di Levante and Ponente. The second zone (Fig. 3c,d) focuses on the promontory of P.ta Aria (southern white inset of Fig. 2). In particular, Fig. 3c,d visualises at a nominal scale 1:1,000 the northern portion of this promontory. The shaded relief (Fig. 3c) reproduces with high detail the rocky coast and a series of aligned features, corresponding to the cultivations well recognizable in the orthoimage of Fig. 3d. In all figures the coastline is visualised in red. Figure 3 well highlights the excellent matching between the coastline derived from the DSM and that visible on the orthoimages.

**Fig. 4:**
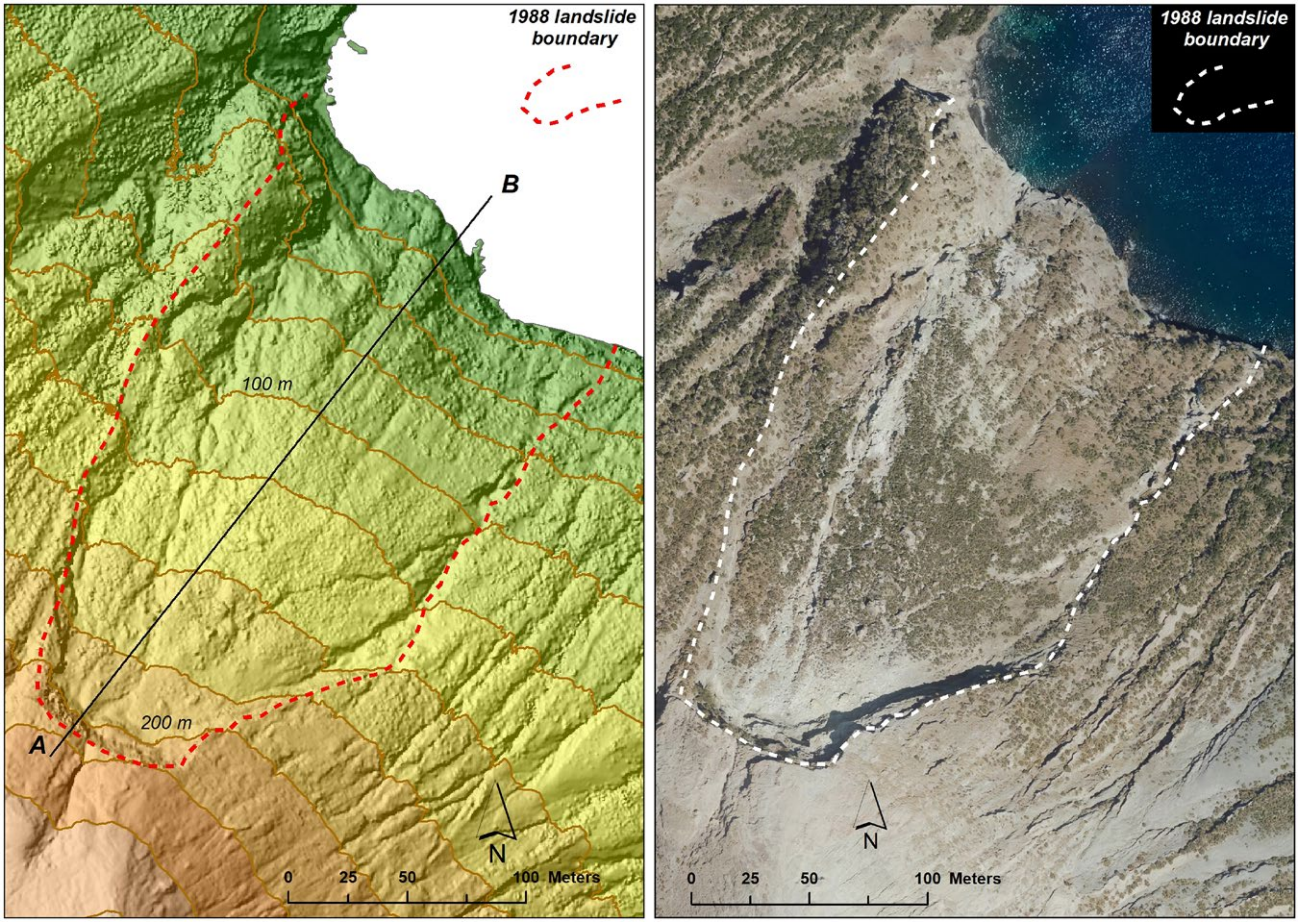
Delineation of the 1988 landslide boundary. The left image shows the Elevation Model overlapped on the Shaded Relief map. The contour lines are reported every 25 meters of elevation. The right image shows that the landslide boundary obtained by the DSM closely matches the landslide limits visible in the orthoimage at spatial resolution of 15 cm. The black line AB indicates the profile trace shown in Fig. 5.

**Fig. 5:**
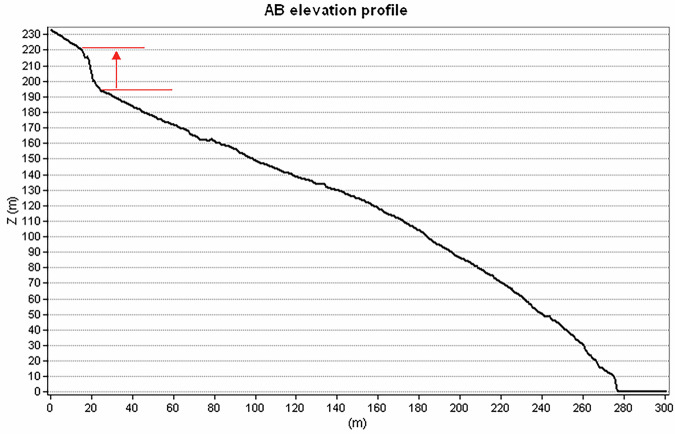
The elevation profile referred to the AB section (Fig. 4) traced along the slope. The red arrow indicates the 29 meters of detachment.

The high resolution of the resulting DSM allows mapping with elevated details also geomorphological features of various interests, such as those induced by earthquakes or extreme rainfalls. A clear example of such phenomena is the landslide that affected the northeastern flank of La Fossa cone (red inset in Fig. 2) on 20^th^ April 1988, probably triggered by the combined effects of high cumulated rainfalls and increased seismicity^25,26^. Figure 4 shows the boundary of the landslide delineated from the DSM and the Shaded Relief map (Fig. 4a). This boundary well matches the limit of the landslide visible on the orthoimage (Fig. 4b). From the DSM, the highest detachment point of the landslide results to be located at an elevation of 225 m a.s.l. (Fig. 5), while the maximum width and length of the landslide are 165 m and 278 m, respectively. In Fig. 5, the detachment scar was analysed along the AB profile, which shows a detachment of 29 meters between 221 m a.s.l. and 192 m a.s.l.

## Data Records

The data record consists of: i) the 3D (x, y, z) points^27^ acquired during the Lidar survey, ii) a DSM^28^ at a spatial resolution of 50 cm, iii) the coastline^29^ of the Vulcano island. The elevation model was obtained by elaborating the elevation points on the ESRI platform (ArcGIS Map 10.5). The coastline was derived from the Elevation Model through specific procedures implemented on the same GIS platform. The 3D points are in ellipsoid heights while the DSM is in orthometric heights. All data are geocoded into WGS 84 (ETRF2000) UTM 33 N Coordinate System. The model, stored in ASC file format, the coastline, stored in polygon shapefile (ESRI format), and the 3D points, stored in csv format, are available within the INGV repository. The link is 10.13127/vulcano/vuldsm2023. All data are shared under the CC BY 4.0 use license.

## Technical Validation

The resulting DSM of Vulcano island has been validated by using a dataset of 17 Ground Control Points (GCPs) acquired by a dedicated Global Navigation Satellite System - Real Time Kinematics (GNSS-RTK) field campaign carried out on 7^th^ February 2024 and data derived from 3 permanent stations belonging to the Vulcano GNSS monitoring network, managed by INGV-OE and acquired during the Airborne Lidar survey (Fig. 1). During the field campaign, 17 GCPs were collected on anthropic and natural features considered stable over time and space, and clearly identifiable both in the field and on the DSM itself. The acquisition of the 17 GCPs was performed trying to ensure a homogeneous spatial coverage of the entire island, excluding areas not accessible due the high steepness or the presence of toxic gas emissions, such as La Fossa cone, or areas where no stable points clearly identifiable both in the field and in the orthoimages were present. The GCPs campaign was carried out using a Trimble R6 L1/L2 receiver, with differential corrections provided by the Spektra Network Real Time Kinematics (NRTK) local area network. The GNSS permanent stations used in this validation are IVLT, IVUG and VCSP. All stations are equipped with LEICA AR20 antennas. Regarding the receiver, IVLT and IVUG stations use LEICA GR50, while the VCSP station uses the LEICA GR20. All instruments acquired the 3D position of the points in latitude, longitude and ellipsoid height. Subsequently, the angular coordinates were converted into metric coordinates (WGS 84 - ETRF2000 UTM 33 N) and the ellipsoid heights were transformed into orthometric heights by using the ConVERGO software.

Table 1 reports for each GCP, the name, latitude, longitude, ellipsoid height, metric coordinate in X, metric coordinate in Y, orthometric height, and the description of the acquisition site.

**Table 1:** GCPs collected during the GNSS-RTK field campaign and retrieved from the permanent GNSS stations.

GCP name	lat (°)	long (°)	ellips. height (m)	X (m)	Y (m)	ortho-Z (m)	Site description
A	38°24′55.2492″	14°57′38.8220″	44.747	496576.440	4251900.995	1.535	Corner of a metal plate
C	38°25′06.4212″	14°57′26.1381″	45.361	496269.015	4252245.478	2.415	Corner of a wall
D	38°25′08.5602″	14°57′08.6051″	44.070	495843.896	4252311.616	0.857	Corner of a metal grid
E	38°24′44.2149″	14°57′00.0000″	58.912	495634.521	4251561.350	15.718	End corner of a small wall
F	38°24′30.3477″	14°57′11.6817″	79.206	495917.902	4251133.785	36.018	Center of a manhole cover
G	38°24′48.1190″	14°57′17.5977″	49.597	496061.646	4251681.461	6.395	Corner of a wall
H	38°22′08.2212″	14°59′39.3150″	44.187	499498.069	4246752.133	1.159	Corner of a metal grid
I	38°22′01.6788″	14°59′30.9816″	49.368	499295.840	4246550.499	6.346	End corner of a wall
J	38°22′32.6036″	15°00′01.8125″	164.398	500043.979	4247503.631	121.349	End corner of a wall
K	38°22′47.9596″	14°59′16.2603″	462.741	498938.800	4247977.004	419.665	Corner of a wall
L	38°23′48.1452″	14°59′15.9703″	416.208	498932.008	4249832.048	373.065	Corner of a stone bench
M	38°23′12.6264″	14°58′59.2619″	413.154	498526.525	4248737.349	370.046	End corner of a wall
N	38°22′58.9658″	14°58′25.4305″	382.660	497705.675	4248316.492	339.559	End corner of a wall
O	38°23′05.0187″	14°57′47.4803″	402.764	496785.047	4248503.372	359.655	Corner of a metal plate
P	38°23′27.3402″	14°57′57.7401″	342.596	497034.206	4249191.270	299.451	Corner of a stone bench
Q	38°23′56.0477″	14°56′57.2346″	134.599	495566.944	4250076.768	91.444	End corner of a wall
R	38°24′21.0845″	14°56′46.9273″	147.222	495317.384	4250848.597	104.050	End corner of a wall
IVLT	38°23′46.2644″	14°56′53.5238″	230.560	495476.766	4249775.276	186.021	GNSS network
IVUG	38°23′48.9916″	14°59′11.3162″	413.936	498819.120	4249858.149	369.389	GNSS network
VCSP	38°24′33.9377″	14°57′5.6557″	76.959	495771.814	4251244.511	31.968	GNSS network

Table 2 summarizes the data used to validate and calculate the accuracy of the DSM. For each GCP, the Z value derived from GPS/Permanent Station and DSM, the instrumental error (expressed as module) related to GPS/Permanent Station and Lidar acquisition, Residual and Field error are reported. In detail, Residual represents the difference between the elevation stored in the DSM cell (Z_DSM_) containing the GCP, and the elevation value of the GCP measured on the field (Z_GPS_) or by the permanent station. The Field error considers the errors of centring (±0.5 cm) and levelling (±0.3 cm) referred to GPS antenna positioning on the field^30^.

**Table 2:** The data used for the DSM validation. PS: Permanent Station.

GCP name	Z GPS-PS (m)	Z DSM (m)	Residual (m)	±Z GPS-PS instrum. error (m)	±Z Lidar instrum. error (m)	±Z Field error (m)
A	1.535	1.588	0.053	0.018	0.050	0.008
C	2.415	2.151	−0.264	0.018	0.050	0.008
D	0.857	0.943	0.086	0.019	0.050	0.008
E	15.718	15.707	−0.011	0.019	0.050	0.008
F	36.018	36.059	0.041	0.029	0.050	0.008
G	6.395	6.443	0.048	0.023	0.050	0.008
H	1.159	1.186	0.027	0.019	0.050	0.008
I	6.346	6.401	0.055	0.026	0.050	0.008
J	121.349	121.272	−0.077	0.021	0.050	0.008
K	419.665	419.716	0.051	0.027	0.050	0.008
L	373.065	372.914	−0.151	0.019	0.050	0.008
M	370.046	370.077	0.031	0.021	0.050	0.008
N	339.559	339.618	0.059	0.023	0.050	0.008
O	359.655	359.649	−0.006	0.02	0.050	0.008
P	299.451	299.356	−0.095	0.019	0.050	0.008
Q	91.444	91.478	0.034	0.029	0.050	0.008
R	104.050	104.064	0.014	0.018	0.050	0.008
IVLT	186.016	186.037	0.021	0.015	0.050	—
IVUG	369.389	369.305	−0.084	0.015	0.050	—
VCSP	31.968	31.928	−0.040	0.015	0.050	—

If the GCP lies within 13 cm of the DSM cell boundary, the elevation value (Z_DSM_) is assumed to be the value stored in the adjacent cell. The threshold of 13 cm derives from the instrumental planimetric error of the Lidar sensor at 1 σ.

**Fig. 6:**
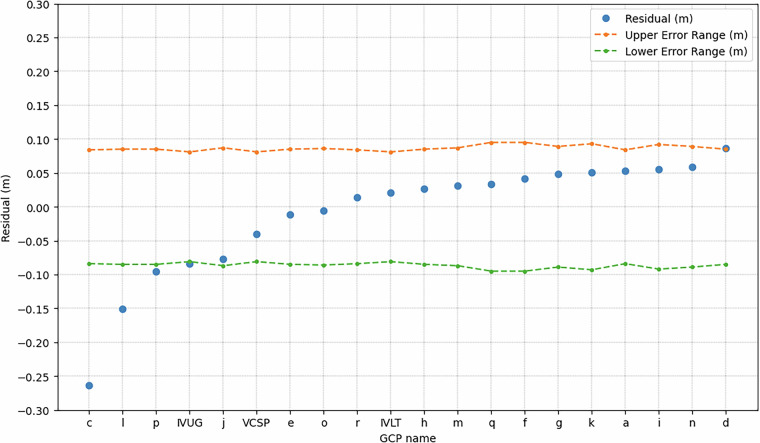
Comparison between Residuals (Z_DSM_−Z_GPS_) and the Total Error for each GCP. The blue points indicate the Residual value calculated for each GCP, the orange and green lines represent the upper and lowest limit of the Total Error range.

The validation of the DSM was performed by comparing, for each GCP, the Residual with the Total Error in Z calculated by summing the instrumental error of GPS, instrumental error of Lidar system and the Field error. The result of this comparison is shown in Fig. 6. The DSM is considered validated if the Residual falls inside the range of the Total Error. This is true for all GCPs except “c”, “l” and “p”(see Fig. 1). Taking into account the “p” residual, it lies at the limit of acceptability as it differs from the lowest total error by only 1 cm. Investigating the other two GCPs, the orthoimages reveal the presence of anthropic features close to “c” and “l” GCPs that probably interfered with GNSS acquisition. From such consideration, it is reasonable to affirm that the DSM is validated by at least 90% of the available GCPs.

The vertical accuracy of the DSM was obtained by calculating the root-mean-square-error (RMSE) of the residuals (Z_DSM_−Z_GPS_) and results in 8 cm. The RMSE value is consistent with the instrumental vertical error (±5 cm) of the Lidar sensor used in the acquisition survey. The validation associated with the calculated accuracy confirms the high quality of the DSM. Thanks to these characteristics, this model represents the most recent and accurate 3D topography of Vulcano island, and for this reason, it can be a very useful tool for land management in case of natural calamities.

## Data Availability

The datasets are archived and shared through the INGV repository. The link is 10.13127/vulcano/vuldsm2023.

## Code availability

No custom code was used to generate or process the data described in the manuscript.
